# The Role of Heat Shock Proteins in the Pathogenesis of Polycystic Ovarian Syndrome: A Review of the Literature

**DOI:** 10.3390/ijms24031838

**Published:** 2023-01-17

**Authors:** Sara Anjum Niinuma, Laila Lubbad, Walaa Lubbad, Abu Saleh Md Moin, Alexandra E. Butler

**Affiliations:** 1 School of Medicine, Royal College of Surgeons in Ireland Bahrain, Busaiteen 15503, Bahrain; 2 Research Department, Royal College of Surgeons in Ireland Bahrain, Busaiteen 15503, Bahrain

**Keywords:** polycystic ovary syndrome, heat shock proteins, therapeutics, insulin

## Abstract

Polycystic ovarian syndrome (PCOS) is the most common endocrine disorder in women of reproductive age and post-menopausal women. PCOS is a multifactorial heterogeneous disorder associated with a variety of etiologies, outcomes, and clinical manifestations. However, the pathophysiology of PCOS is still unclear. Heat shock proteins (HSPs) have recently been investigated for their role in the pathogenesis of PCOS. HSPs are a class of proteins that act as molecular chaperones and maintain cellular proteostasis. More recently, their actions beyond that of molecular chaperones have highlighted their pathogenic role in several diseases. In PCOS, different HSP family members show abnormal expression that affects the proliferation and apoptotic rates of ovarian cells as well as immunological processes. HSP dysregulation in the ovaries of PCOS subjects leads to a proliferation/apoptosis imbalance that mechanistically impacts follicle stage development, resulting in polycystic ovaries. Moreover, HSPs may play a role in the pathogenesis of PCOS-associated conditions. Recent studies on HSP activity during therapeutic interventions for PCOS suggest that modulating HSP activity may lead to novel treatment strategies. In this review, we summarize what is currently known regarding the role of HSPs in the pathogenesis of PCOS and their potential role in the treatment of PCOS, and we outline areas for future research.

## 1. Introduction to Polycystic Ovarian Syndrome

Affecting approximately 6–10% of women of reproductive age, PCOS is the most common endocrine disorder and cause of anovulatory infertility in women [1,2,3]. Diagnostic criteria for PCOS were established and accepted by the European Society of Human Reproduction and Embryology/American Society for Reproductive Medicine (ESHRE/ASRM) in Rotterdam in 2003, and these Rotterdam consensus guidelines are the most widely accepted and commonly used criteria for PCOS diagnosis. For diagnosis, the presence of two out of three diagnostic criteria is required: (1) oligo- or anovulation, (2) biochemical or clinical hyperandrogenism (clinical signs being acne, hirsutism, and androgenetic alopecia), and (3) characteristics of polycystic ovarian morphology (PCOM) as assessed by ultrasound scanning of the ovaries (an ovary with ≥20 follicles, with a diameter of 2–9 mm and/or volume ≥10 cm^3^) [4].

In a healthy female ovary, an egg/ovum is released once a month and will either be fertilized or released during menstruation. In women with PCOS, a variety of factors can prevent the egg from being released from the ovary, which is referred to as anovulation. Antral follicles and immature eggs are arrested at the preovulatory stage, leading to the formation of small cysts in the ovary. Consequently, this results in irregular menstrual cycles and an increased likelihood of infertility [1].

Multiple factors, including disturbances in the release of gonadotropins, cause anovulation in women with PCOS. This is largely due to abnormal regulation of the secretion of hormones by the hypothalamus [5]. A high frequency of luteinizing hormone (LH) pulses leads to the high LH:follicle stimulating hormone (FSH) ratio commonly found in women with PCOS [1]. FSH levels become relatively low, causing follicles in the ovary to become resistant to the hormone. This interferes with normal follicular growth, which is also believed to be due to elevated anti-Mullerian hormone levels [2]; however, the raised LH:FSH ratio is not used for the diagnosis of PCOS [6].

**Figure 1:**
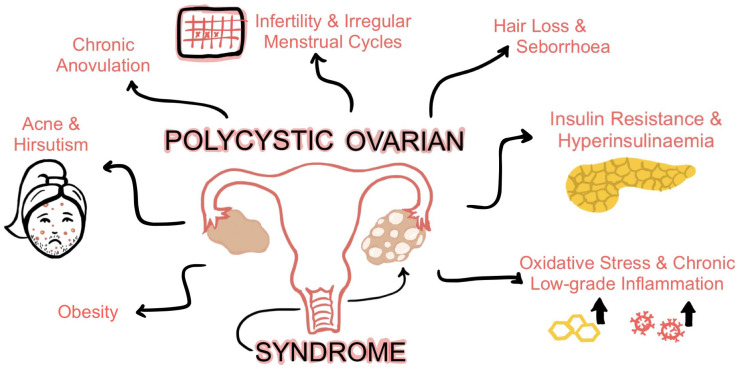
The clinical manifestations of PCOS in women.

PCOS is a heterogeneous disorder clinically manifested by hirsutism, acne, irregular periods, and other features, as illustrated in Figure 1. These symptoms are largely due to increased androgen levels in women with PCOS. Androgens have an inhibitory effect on the production of sex-binding globulin in the liver, leading to acne, hirsutism, and, less commonly, androgenetic alopecia due to elevated circulating testosterone [7]. Additionally, androgens prevent the maturation of the dominant follicle and the apoptosis of smaller follicles in the ovary, which causes the characteristic formation of cysts around the border of each ovary [7]. Estrogen dominance and slightly elevated prolactin levels are also notable reasons underlying the irregular menstrual cycles, mood disturbances, headaches, bloating/weight gain, and infertility [8]. Other major manifestations of PCOS, in particular insulin resistance and hyperinsulinemia, also play a role in the folliculogenesis and maintenance of hyperandrogenemia. Elevated circulating insulin stimulates LH to increase androgen formation in ovarian theca cells [2]. This leads to reproductive and metabolic abnormalities and increased inflammation in women with PCOS.

Several factors play a role in the pathophysiology of PCOS, a key one being oxidative stress, an imbalance in the level of oxidants and antioxidants in the body [7]. In PCOS, this imbalance occurs due to a combination of metabolic disturbances in the body, including hyperinsulinemia, dyslipidemia, hyperglycemia, and obesity (which has a prevalence of 30–40% in women with PCOS) [7]. Each of the above-mentioned conditions leads to a buildup of reactive oxidative species (ROS), causing an imbalance in their concentrations and resulting in dysregulation of cell growth, differentiation, and apoptosis. ROS also stimulate the production of inflammatory markers such as tumor necrosis factor alpha (TNF-α) and nuclear factor kappa-light-chain-enhancer of activated B cells, both of which cause an increase in androgen production and reinforce the development of symptoms caused by hyperandrogenism [7]. Additionally, TNF-α plays a role in stimulating insulin resistance, exacerbating the underlying metabolic irregularities that may augment the progression of PCOS [7]. An increase in lipids in the endometrium of obese women with PCOS has also been found to have an additive inflammatory effect due to disruption of homeostasis in the endometrial endoplasmic reticulum (ER) [9]. This leads to an ER stress response, impacting the normal physiological function of endometrial cells and increasing the risk of PCOS pathogenesis progression [10].

PCOS patients have distinct changes in gene expression in their ovarian tissues [11]. One study reported 573 upregulated and 430 downregulated genes in the ovaries of women with PCOS [11]. The dysregulated genes were notably involved in the metabolism and biosynthesis of lipids, cholesterol, and steroids [11]. However, it is still unclear whether these dysregulated genes are directly associated with the pathogenesis of PCOS [11] as gaps remain in the current understanding of the pathophysiological mechanisms underlying PCOS. Due to the uncertainty about the etiology of this condition, a standardized treatment approach has yet to be agreed upon. One current area of interest is the role of HSPs in the pathogenesis of PCOS and their potential as therapeutic targets for the treatment of PCOS.

## 2. Biology of Heat Shock Proteins

**Figure 2:**
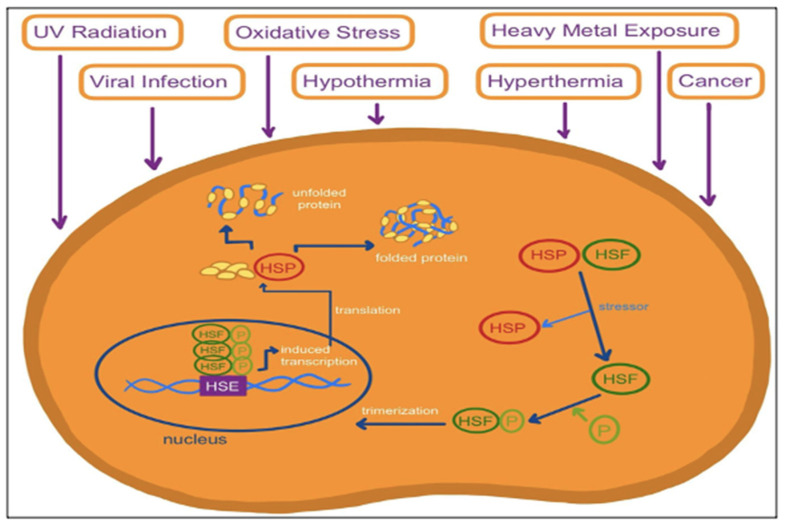
The molecular mechanism for the synthesis of heat shock proteins (HSPs). Under physiological conditions, an HSP and a heat shock factor (HSF) are a complex found in the cytosol in an inactive state. However, stressors such as UV radiation, viral infection, heavy metal exposure, oxidative stress, hypothermia, hyperthermia, and cancer cause HSFs to become detached from HSPs. HSFs undergo phosphorylation and trimerization. Once inside the nucleus, they bind to heat shock elements (HSEs), initiating transcription. The HSP mRNAs then leave the nucleus and are translated, synthesizing new HSPs. These HSPs then act as molecular chaperones and bind to polypeptide chains to prevent misfolding of proteins. To protect cells, HSPs also repair and refold damaged proteins under stressful conditions.

Heat shock proteins (HSPs) are a group of proteins discovered in 1962 whose expression is triggered by heat shock or other stressors [12]. HSPs are classified and named based upon their size and molecular mass [13]. They range from 10 to 150 kDa and are expressed in all major cellular compartments [13]. HSPs are a ubiquitous group of molecular chaperones that bind to polypeptide chains to prevent protein misfolding [13]. They are expressed at low levels when the cell is in its normal physiological state but become elevated once a cell faces a stress stimulus [14]. Figure 2 illustrates how stress conditions cause HSPs to be activated and synthesized. HSPs function mainly to protect against cellular stress [15]. HSPs are found in endothelial cells and play an essential role in cell function modulation and protein homeostasis [14,15]. They are also involved in protein homeostasis functions such as protein folding, degradation, and assembly [16].

In metabolic disorders, cells undergo various stresses, among which genotoxic, oxidative, and metabolic stresses are well documented; however, emerging evidence has revealed proteotoxic stress (perturbations of protein synthesis, misfolding of proteins) to be a common feature of metabolic syndrome [17], features of which are very common in PCOS, and hence, changes in HSPs are not unexpected [18]. Proteostasis (or proteome homeostasis) is a process by which cells balance the processes of protein biosynthesis, folding, and degradation, which are vital to cellular fitness. To counter proteomic perturbations and preserve proteostasis, cells activate the protective response (termed the heat shock response (HSR)) by mobilizing the evolutionarily conserved transcriptional program that is orchestrated in eukaryotic cells by heat shock factor 1 (HSF1) [19]. The HSR is a cellular tool to cope with different stressors, including heat, cold, and metabolic stresses [20], and is mediated by HSF1-dependent heat shock proteins (HSPs). HSPs function as molecular chaperones that recognize and bind to other proteins when these other proteins are in non-native conformations. HSPs function as a facilitator of native protein stabilization, translocation, re-folding, and degradation when they are under proteotoxic stress due to protein denaturation or because the peptides that comprise them have not yet been fully synthesized, folded, assembled, or localized to an appropriate cellular compartment [21].

HSPs have been classified into six major families according to their molecular size: HSP100, HSP90, HSP70, HSP60, HSP40, and small heat shock proteins. The HSP70 family consists of two sub-classes; one is constitutively expressed (HSC70, 73-KDa) and the other is stress-inducible HSP 70 (HSP 72-KDa). Under normal physiological conditions, HSP72 is expressed at low levels; however, following stress stimuli, intracellular synthesis and release of HSP72 increases markedly to function as both an intracellular and extracellular chaperone in cytoprotection [22]. Likewise, the 90-KDa HSP (HSP90) consists of two isoforms, namely inducible HSP90α and constitutively expressed HSP90β [23]. Other large HSPs include the HSP40s or J-proteins, which interact with HSP70 through their J domain and serve as regulatory co-chaperones [24]. Among many aspects of dealing with misfolded proteins, HSPs function in the proteolysis of substrates by the proteasomes. HSP70 and/or HSP90 and related co-chaperones are required for the recognition of misfolded or mislocalized proteins that are subsequently degraded by the proteasome [25,26]. The exact mechanism linking the chaperone and proteasomal systems is still under investigation; however, in different cellular models, HSP70 and its co-chaperone DNAJ were also found to be required for proteasomal degradation of misfolded proteins [25,26].

While classically elevated due to heat shock, HSPs can also be produced in excess under other stressful conditions such as UV radiation, viral infections, oxidative stress, and heavy metal exposure [27,28]. Under certain conditions, a cell can prioritize the HSP response over autophagy and the HSP system can prioritize itself over autophagy if both systems are activated [27,28]. Since most HSPs are expressed under stress, they are considered highly sensitive disease biomarkers [16].

Recent reports have linked HSPs to a variety of pathological conditions. For example, they are overexpressed in many cancers [29]. They are involved in cancer cell development because HSPs inhibit programmed cell death, which is an essential component of the normal cell cycle [30]. HSPs do this by suppressing p53, a protein commonly inactivated in cancers [28]. HSP90B1 has also been reported to play a crucial role in suppressing autophagy and cell apoptosis [11,30]. In addition, high levels of bacterial HSPs, such as HSP60 and HSP70, are involved in many diseases, including type 1 diabetes, Crohn’s disease, atherosclerosis, and juvenile chronic arthritis [13]. HSPs have been reported to be expressed as autoantigens in atherosclerosis development and expressed in cardiovascular diseases where they induce inflammation [31].

Similarly, recent evidence indicates that HSPs play a role in the pathogenesis of PCOS. For example, HSP70 is an essential family of molecular chaperones with multiple functions that include regulating cell-mediated immunity, apoptosis, and cellular stress responses [17,32]. Elevated HSP70 levels have been found in non-obese PCOS patients [33]. HSP70 expression is also correlated with insulin resistance and is involved in the ovarian stress response [33]. Increased levels of HSP70, together with insulin resistance, oxidative stress, and low-grade chronic inflammation, have been found in PCOS patients [10]. HSPs and reproductive hormones closely correlate, which indicates in part how HSPs are involved in PCOS pathogenesis [33].

This review aims to put into context the existing evidence on the association between HSPs and PCOS, particularly their role in the pathogenesis of PCOS and its related conditions, and discusses the potential use of HSPs as therapeutic agents for the treatment of PCOS.

## 3. The Effects of Heat Shock Proteins on the Pathogenesis of Polycystic Ovarian Syndrome

**Figure 3:**
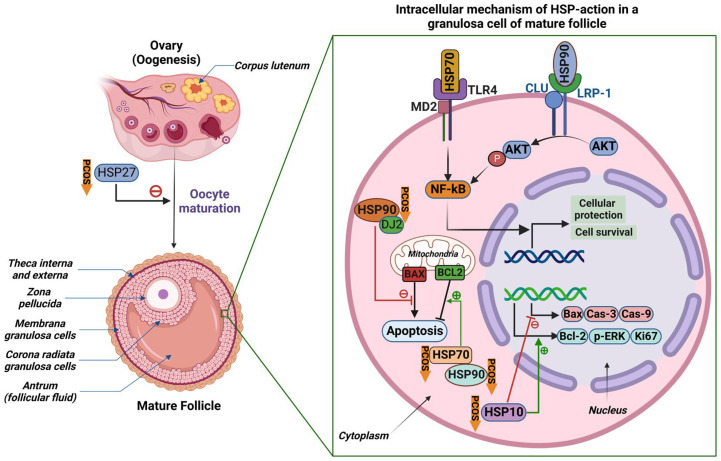
The effect of heat-shock proteins (HSPs) on the pathogenesis of polycystic ovary syndrome (PCOS). (**Left panel**) The stages of oogenesis in the ovary and the structure of a mature follicle. HSPs function at different stages of oogenesis in PCOS. HSP27 negatively regulates oocyte maturation and was downregulated in women with PCOS. (**Right panel**) Intracellular mechanisms of action of HSPs in a granulosa cell (shown in a small green square) of a mature follicle. HSP70 binds to the cell surface toll-like receptor 4 (TLR4) and co-receptor myeloid differentiation protein 2 (MD2). HSP90 binds to the cell surface low-density lipoprotein (LDL)-related protein-1 (LRP-1) in association with clusterin (CLU). Downstream signaling of both HSP70 and HSP90 (phosphorylation of protein kinase B, AKT) leads to the activation of NF-ĸB, a novel transcription factor that regulates the transcription of genes related to cell survival and protection. HSP90 and its co-chaperone DJ2 (HSDJ/hdj-2/rdj1) inhibit mitochondrial proapoptotic Bcl-2 associated X-protein (BAX). HSP90, in association with HSP70, potentiates the mitochondrial antiapoptotic protein B-cell lymphoma 2 (BCL2). HSP10 regulates the transcription of multiple proteins related to cellular apoptosis. Cas-3, caspase-3; Cas-9, caspase-9; p-ERK, phosphorylated extracellular signal associated kinase; Ki67, marker of proliferation Ki-67. Downward orange arrows labeled “PCOS” indicate the HSPs that were found to be downregulated in PCOS in previous publications. This illustration was created using biorender.com (with publication license).

Expression levels of HSPs are altered in the ovarian tissues of women with PCOS and can affect PCOS pathology via different mechanisms (Figure 3). Their expression has been particularly associated with apoptosis and cell viability. A study in 2016 found an increase in HSP90B1 of at least 2-fold in the ovarian tissues of women with PCOS in comparison to normal subjects [11]. HSP90 isoforms have been associated with epithelial ovarian cancer, cancer cell survival, apoptosis, and circadian clock gene expression [34,35]. Reduced HSP90 levels may also affect cell survival; for example, decreased expression of HSP90 was found to be associated with reduced zona pellucida thickness and oocyte growth in mouse zygotes [36]. On this basis, the 2016 study investigated the role of HSP90B1 in the pathogenesis of PCOS. Knockdown of HSP90B1 levels was found to decrease cell viability and increase apoptosis in ovarian cells of women with PCOS [36]. On the contrary, cell survival was increased, and apoptosis of ovarian cells was decreased by at least 50%, in ovarian cells overexpressing HSP90B1 [36]. PCOS has been linked to an increase in granulosa cell proliferation [37]. This study suggested that HSP90B1 may play a role in granulosa cell proliferation and ovarian cell survival and is, consequently, highly likely to be involved in the pathogenesis of PCOS.

HSP70, the most conserved protein, is also increased in ovarian tissue in women with PCOS and was associated with a decrease in apoptosis of ovarian follicular cells. Velázquez et al. found the most intense HSP70 immunostaining in the granulosa and theca cells of cystic follicles of rats with induced PCOS compared to other follicular cell types [38]. In a study in a dehydro-epiandrosterone (DHEA)-induced PCOS rat model, HSP70 was found to be significantly increased in ovarian tissues of the PCOS versus the control group, but also significantly reduced in serum [33]. This increase in HSP70 may contribute to apoptosis in the ovarian tissues of women with PCOS because HSP70 and its co-chaperone, dj2, can affect apoptosis by preventing the Bcl-2 family protein, Bax, from translocating from the cytosol to the mitochondria [39].

The imbalance between apoptosis and antiapoptosis in the ovaries of patients with PCOS has also been demonstrated in studies focused upon HSP10. Downregulation in HSP10 expression in human granulosa cells of PCOS versus normal ovaries has been reported [40]. In a recent study, mouse granulosa cells demonstrated low levels of HSP10 when injected with testosterone and displayed poor viability, suggesting that HSP10 is a downstream factor in ovarian granulosa cell apoptosis [41]. Lower expression of HSP10 was associated with the upregulation of proapoptotic factors such as Bax, caspase-9, and caspase-3 [41]. Reduced expression of HSP10 was associated with the downregulation of the antiapoptotic factors Bcl-2, phosphorylated-ERK, and Ki67 [41]. Overexpression of HSP10 demonstrated opposite effects [42]. Hyperandrogenic conditions lowered HSP10 expression and consequently induced apoptosis in mouse granulosa cells [41].

Similarly, proteomic analysis has shown that small HSPs, such as HSP27, an antiapoptotic protein, are downregulated in the oocytes of women with PCOS [40]. In a study on mouse oocytes, downregulation of HSP27 improved oocyte maturation and led to early-stage apoptosis, thus countering the atretic follicles found in PCOS [43]. To further elucidate the role of HSP27 in oocyte development, a study employing overexpression of HSP27 found a reduced oocyte maturation rate and reduced expression of apoptotic-related factors in women with PCOS [44]. This scenario can lead to the development of multiple small antral follicles [44]. These HSP27-induced mechanisms can play a role in the altered fertility of patients with PCOS as lower expression of HSP27 can lead to abnormal oocyte development, characteristic of PCOS. Other small HSPs, for example α-crystallin, have also been found to be associated with endocrine disorders in women. Elevated levels of anti-α-crystallin antibodies were found in patients with PCOS, due to the overproduction of α-crystallin in response to oxidative stress and chronic inflammation in PCOS [45].

Taken together, these findings indicate that the rate of granulosa cell proliferation and apoptosis is affected in women with PCOS and can lead directly to the development of PCOS. Das et al. observed fewer apoptotic granulosa cells and increased proliferation rates in the PCOS anovulatory follicles versus normal ovulatory follicles [37]. Furthermore, activated caspase-3, a functionally required protein for granulosa cell apoptosis, was found to be significantly reduced [37]. In addition, increased gene expression of inhibitors of apoptotic proteins (IAP) and Bcl-X_Long_ (an antiapoptotic factor) and downregulated expression of Bax (proapoptotic) were reported [37]. By contrast, lower proliferation rates were reported in granulosa follicles of rats with induced ovarian cysts [46]. These results are consistent with other animal studies [42,47]. However, an increase in proliferation can be attributed to the high levels of androgens that occur in human PCOS, but not in induced animal models. The evidence is also conflicting, since other studies on DHEA-induced PCOS rats found apoptosis of granulosa cells to be increased and an imbalance of Bcl-2 family members [48]. Moreover, it should be noted that the unknown nutritional conditions and other stress factors of the animal models used in the studies could also influence the expression of HSPs and thus affect the results. Regardless, there is strong evidence that an imbalance between apoptosis and proliferation can serve as a mechanism to impact follicle development and lead to polycystic ovaries. HSPs appear to play a role in this aberrant folliculogenesis in women with PCOS.

HSPs can also lead to immunological abnormalities in PCOS. This is of significance since the breakdown of immune homeostasis has been linked to the pathogenesis of PCOS [5]. In a cross-sectional study, the significantly higher levels of HSP70 in the PCOS cohort were correlated with the regulatory T cell (Treg)/T helper cell 17 (Th17) ratio [32]. Th17 cells produce an inflammatory autoimmune response, whereas Treg cells inhibit this inflammatory phenomenon. In this way, these two cells balance each other functionally and maintain homeostasis of the immune system. Serum HSP70 levels were negatively correlated with the Treg/Th17 ratio, indicating that high levels of HSP70 promote the expression of the Th17 gene [32]. This may be because of HSP70’s ability to enhance the immune response under stress. Blocking the function of HSP70 can lead to a reduction in the inflammatory response in women with PCOS [32]. This demonstrates that HSPs may not only change their expression profiles in PCOS and affect the rates of proliferation and apoptosis, but also affect the immunological pathogenesis.

The factors regulating the HSPs in PCOS have not been fully elucidated. However, emerging evidence suggests that non-coding RNAs (ncRNAs) may play an important role in heat responses and the regulatory mechanisms of HSPs. The ncRNAs (which do not encode a protein) are a class of regulatory RNAs including microRNAs (miRNAs), small interfering RNAs (siRNAs), long non-coding RNAs (lncRNAs), and circular RNAs (circRNAs). Among ncRNAs, lncRNAs have been reported to be involved in the regulation of the heat stress (HS) response in some species [49,50,51,52]. The “non-coding” part of the lncRNA may be involved in the rapid and coordinated reshaping of protein-coding gene expression [53]. The mechanism of lncRNA actions in regulating HS may involve interaction with the transcription factor heat shock transcription factor 1 (HSF1) [54] or feedback regulation of key stress response proteins encoded by the pseudo-gene of HSP70 [55,56]. In a well-established HS-rat model, potential functions of lncRNA as transcriptional regulators of the HS response have been found in the liver and adrenal glands [57], indicating the involvement of lncRNAs in regulating HSPs in a wide variety of tissues. Previous research has demonstrated the significant differential expression of small ncRNAs (sncRNAs) in serum, granulosa cells (GCs), follicular fluid (FF), and other tissues between women with PCOS and healthy control women [58,59]. Moreover, the dysregulation of lncRNAs in PCOS and the potential influence of lncRNAs in the pathogenesis of PCOS have also been reported [60]. Taken together, it is tempting to speculate that, in PCOS, the molecular mechanisms of heat stress responses (mediated by HSPs) involve non-coding RNAs (ncRNAs).

The evidence for the role of HSPs in the pathogenesis of PCOS is summarized in Table 1. Moreover, HSPs may also play a part in the pathogenesis of conditions associated with PCOS, such as diabetes and obesity.

## 4. The Role of Heat Shock Proteins in Type 2 Diabetes

The risk of T2D is significantly increased in women with PCOS [61], to the extent that PCOS has been identified as a significant non-modifiable risk factor associated with T2D by the International Diabetes Federation [62] and by the American Diabetes Association [63]. Literature regarding the involvement of HSPs in the mechanism of PCOS and diabetes exists but is limited. For example, HSP70 serum levels were increased in women with PCOS, and this was associated with insulin resistance (IR), low-grade chronic inflammation, and oxidative stress, indicating that HSP70 could potentially serve as an independent marker for PCOS [14]. HSP70 levels were higher in patients with diabetes versus controls, especially in patients who had diabetes for longer than 5 years [64]. HSP70 leads to IR and may lead to diabetes, but this relationship seems to be bidirectional; IR can also facilitate the accumulation of HSP70 in the body [65]. This provides evidence for a link between HSP70 and IR. However, conversely, a study of Caucasian subjects with T2D found reduced expression of the HSP70 gene [66]. A study on mice also found increased expression of the related HSP72 that protects against insulin resistance by preventing the phosphorylation of inflammatory signaling proteins [67].

Other HSPs have been implicated in the pathogenesis of diabetes. HSP40/DNAJB3 was significantly decreased in obese and diabetic subjects, and this expression was upregulated with a regular exercise protocol [68]. Diabetic mice had reduced expression of HSP60 in the brain, which was suggested to cause mitochondrial dysfunction and IR in the hypothalamus [69]. The expression of heat shock factor protein 1 was also altered in the islets of rat models of spontaneous T2D [70]. These results indicate that HSPs are involved in insulin resistance, but there are minimal studies on the direct involvement of HSPs in the pathogenesis of PCOS women with diabetes.

Similarly, gaps in the literature exist for the role of HSP72 in the development of diabetes. The expression of intramuscular HSP72 was reduced in patients with T2D [71], and this decreased expression has been inversely correlated with IR [66]. The low expression can contribute to a higher risk of IR and, consequently, diabetes.

Furthermore, reduced HSP72 expression during endoplasmic reticulum stress can lead to beta cell dysfunction and reduced insulin secretion [72]. This is of concern since HSP72 is reduced in women with obesity, and obesity is common in women with PCOS [67]. Mice lacking HSP72 were phenotypically obese and had insulin resistance in skeletal muscle and glucose intolerance [73]. Even in healthy individuals, reduced levels of HSP72 in skeletal muscle were associated with decreased insulin sensitivity and increased adiposity [74]. However, a recent study on T2D mice and humans did not find HSP72 to be reduced in T2D, nor did it find reduced HSP72 to be associated with IR in T2D [75]. This conflicting evidence warrants further research with regard to the role of HSPs in T2D and PCOS.

## 5. The Role of Heat Shock Proteins in Obesity

Obesity is another common finding in women with PCOS. Anywhere from 38 to 88% of women with PCOS are overweight or obese [76]. Even moderate weight loss can result in clinically beneficial improvements in the metabolic features of PCOS [76]. In women with PCOS, saturated fat ingestion can stimulate a proatherogenic inflammatory response, even in non-obese women with PCOS [77]. This response is enhanced by the combination of PCOS and obesity [78]. In a study on women with PCOS, ingestion of saturated fat led to a more significant increase in serum HSP70 levels in lean women with PCOS compared to lean controls and in obese women with PCOS compared to obese controls [78]. HSP70 was one of the proatherogenic inflammatory markers identified in this study; however, the inflammatory response was more significant in the setting of PCOS and obesity combined versus obesity alone [78]. In addition, HSP70 was inversely correlated with insulin sensitivity [78]. This suggests a mechanism through which women with PCOS are susceptible to atherogenesis [78].

Other studies have also linked HSPs to obesity, but research using PCOS samples is limited. HSP72 can reduce inflammation and prevent insulin resistance induced by diet or obesity [67]. However, obesity was linked with the reduced expression of HSP72 and increased JNK phosphorylation in skeletal muscle [67]. Intracellular HSP70 and HSP27 miRNA expression levels were significantly reduced in obese subjects with metabolic syndrome compared with obese subjects without metabolic syndrome [77], a finding applicable to PCOS since metabolic syndrome is present in 33% of women with PCOS [79]. Serum HSP60 levels were higher in obese versus lean subjects, and bariatric surgery reduced circulating HSP60 [80]. These studies provide evidence that HSPs may play a role in the pathogenesis of obesity, but further studies are necessary to investigate its role in women with PCOS with stratification of lean, overweight, or obese body mass indices.

## 6. Therapeutic Interventions

Due to the complex and largely unknown pathogenesis of PCOS, researchers have not yet been able to pinpoint an exact cause, nor have they found a definitive treatment for the condition. However, HSPs play a significant role in the progression of this multifactorial disorder. Abnormal levels of HSPs are a common finding in women with PCOS, and it has been established that they are involved in many aspects of the condition, making them suitable targets for possible treatments. Nevertheless, in searching for an effective way to modify HSPs, the reported evidence is contradictory. This suggests that HSPs cannot broadly be labeled as “good” or “bad”, but simply integral factors in the mechanism of PCOS that, once disrupted, contribute to its pathogenesis and can potentially be modulated to alleviate the condition. In the following sections, we explore the various evidence-based therapies for PCOS and their association with HSPs.

### 6.1. Induction of Heat Shock Proteins through Exercise

Following diagnosis, exercise and lifestyle are first-line therapies advised for women with PCOS. This approach is mainly associated with increased HSPs in the body in response to stressors such as exercise. PCOS is an inflammatory disorder, and HSPs are known to be induced following stressors such as inflammation and oxidative stress [81]. HSP70 and HSP72 can block heat-induced apoptosis, which occurs through SAPK/JNK stimulation [82]. These proteins inhibit steps that occur upstream and play a role in minimizing the cleavage of poly (ADP-ribose) polymerase, a common death substrate protein [82]. Exercise induces the expression of HSPs, which would increase the inhibition of apoptosis and molecular damage as a result of disturbances in cell homeostasis in inflammatory states [83].

In one study, an 8-week moderate-intensity exercise program was performed to explore the effects of exercise on inducing the expression of HSPs and determine if this process is efficient in women with PCOS versus controls [15]. By the completion of the experiment, both groups showed some improvement in VO_2_ max, waist–hip ratio, BMI, and systolic blood pressure; however, these results were seen to a much lesser extent in the PCOS cohort. As for HSP72 expression in monocytes and lymphocytes, the control group showed a significant increase, while the increased trend in the PCOS cohort was not significant [15]. Thus, it was concluded that the HSP response to exercise was impaired in women with PCOS compared to the control group, and it was suggested that high-intensity exercise could have more favorable effects in inducing HSP activity [15]. It is, however, important to note that the small sample size, inability to evaluate the influence of obesity, and failure to measure serum HSP levels could have affected the outcomes of this study.

Further research into alternative methods to effectively induce HSPs, such as high-intensity interval training and resistance training, could have therapeutic implications for women with PCOS. Weightlifting has also been shown to be suitable for women with PCOS because it decreases testosterone levels, lowers IR, and, most importantly, increases metabolic rate [84]. More extensive studies are needed on this treatment approach as the exact association of these approaches with HSPs has not been adequately investigated.

### 6.2. Heat Shock Therapy

The role of heat shock therapy could be promising for patients with PCOS. A study has shown that heat therapy decreases sympathetic activity and enhances cardiovascular health in obese women with PCOS [85]. Heat therapy comprises several approaches, such as using a sauna, hot tub, and combined exercise heat stress [86]. This could be a promising approach since it has been clinically proven to improve cardiometabolic health in obese people [85]. Since obesity and PCOS both have potential cardiovascular risks, exploring and comparing heat shock therapy in both pathological conditions would be of interest.

It is important to note that there are not many pharmaceutical or lifestyle interventions that would decrease the cardiovascular risk for women with PCOS [85]. Most research and treatment options aimed at decreasing cardiovascular risk target men, thus it is crucial to further explore options and therapeutic interventions that can help women who have cardiovascular risk factors, including women with PCOS.

One study has found that, through a program of 30 one-hour heat therapy sessions conducted on obese women with PCOS, there were improvements in carotid and femoral artery wall thickness and decreased total cholesterol and fasting glucose [85]. Heat therapy clinically improved indicators of PCOS, such as lowering serum testosterone and normalizing menstrual cycles [87]. This emphasizes the role of heat therapy in metabolic and ovarian function in women with PCOS [87]. Another study showed that heat therapy in obese women with PCOS improved PCOS symptoms, systemic insulin sensitivity, whole-body glucose uptake, and insulin signaling in subcutaneous adipose tissue [87]. A single heat treatment lasting 20 min increased HSP72 levels maximally in all depots of white adipose tissue and also improved insulin signaling [88]. The beneficial improvements due to heat therapy can be attributed to increased levels of HSPs which act to inhibit inflammatory factors such as JNK [88]. Low levels of HSPs were found in adipose tissue of T2D patients, further supporting the association between increased levels of HSPs and metabolic health [89].

Nonetheless, these studies have limitations as they only included obese women with PCOS. Future research regarding heat therapy in non-obese women with PCOS remains to be explored. Further studies are also needed to analyze the effects of chronic heat treatment on the activity of HSPs.

### 6.3. Induction of HSPs to Treat Insulin Resistance

Although limited research has been conducted on the direct use of HSPs as therapy for PCOS, HSPs have been proven to combat insulin resistance. Patients with T2D were shown to have reduced gene expression of HSP72 [67]. According to a model proposed by Hooper and Hooper (2009), this decrease in HSPs is due to insulin resistance stimulated by inflammation in obesity [15]. When fatty acid levels are elevated in muscle fibers, this stimulates the formation of diacylglycerol and the activation of protein kinase C [90]. This leads to the phosphorylation of a critical residue on insulin receptor substrate-1 by IKK-beta and JNK, preventing the substrate from effectively interacting with the active insulin receptor [90]. HSP27 and HSP72 can prevent the activation of IKK-beta and JNK, thus allowing IRS-1 to appropriately bind to the receptor.

The use of bimoclomol and lipoic acid, inducers/co-inducers of HSPs, in obese rodents with insulin resistance was proven to improve insulin sensitivity [90]. BPG-15, another co-inducer of HSPs, was tested on insulin-resistant patients versus placebo and resulted in a notable increase in insulin sensitivity and glucose utilization [91]. This hypothesis was also tested on human subjects using heat shock therapy, transgenic overexpression in rodent models, and pharmacological drugs to induce HSP72 locally in skeletal muscle (as JNK phosphorylation was thought to be increased in skeletal muscle) [67]. Data showed that the increased expression of HSP72 prevented the phosphorylation of JNK and thus decreased diet/obesity-induced hyperglycemia, hyperinsulinemia, glucose intolerance, and insulin resistance [67].

Another study further tested the effects of HSPs using mild electrical stimulation (MES) in combination with heat shock [92]. Different doses and durations were tested in vivo and in vitro. This combined treatment for 10 min, twice weekly, could lower insulin resistance, insulin levels, and fasting blood glucose. In mice, this treatment stimulated the insulin signaling pathway and thus improved fat metabolism [92]. HSP70 was also shown to protect against oxidative stress injury in diabetes and insulin resistance. This action is stimulated by the reduced release of nitric oxide, which leads to inflammation and simultaneously induces HSP70 in an attempt to reduce it [93]. Considering that insulin resistance is a major symptom of PCOS, treating this aspect alone would undeniably improve patient quality of life. However, additional research exploring the effects of HSP induction on women with insulin resistance and PCOS is needed to ensure that these findings have therapeutic relevance.

### 6.4. Repression of Heat Shock Proteins through miRNAs

Although HSPs are generally believed to have a protective role, there is evidence suggesting that HSPs play a role in exacerbating the progression of PCOS [94]. MicroRNAs play a significant role in various diseases, such as cancers, metabolic disorders, and PCOS. HSP function is regulated by differing miRNAs, and hence, they have been a common target for experimental cancer therapies [95]. Researchers have applied the same methodology to PCOS because of the known involvement of miRNAs in the condition. The presence of miRNAs in follicular fluid allows them to influence follicular development and regulate dysfunction in granulosa cells that could otherwise lead to abnormalities such as PCOS [94]. An experiment conducted on DHT-induced rats revealed that 24% of potential miRNAs were differentially expressed in the PCOS group versus controls [94,96]. Another study used an RNase III enzyme necessary for the biogenesis of miRNAs, Dicer 1, to showcase their effects on ovarian function [5]. Knockout of Dicer 1 in mice led to infertility, decreased ovarian weight, decreased ovulation, disrupted the formation of the corpus luteum, and increased synthesis of cysts on the oviducts [5].

Due to the prevalence of miRNAs in ovarian function and their ability to influence heat shock activity, a study investigated the use of miR-144-3p to reduce HSP activity and whether that would yield favorable results in women with PCOS [94]. Initially, decreased expression of miR-144-3p and increased HSP70 expression were found in women with PCOS [94]. The miRNA was then upregulated/downregulated to measure its effects on HSPs. Upregulation of miR-144-3p led to stimulation of granulosa cell survival/proliferation and repressed cell apoptosis. Furthermore, an inhibitory effect on serum testosterone, estrogen, and LH levels was observed, and FSH was increased. Downregulation of the protein had opposite effects and contributed to the progression of endocrine disorders and ovarian weight. HSP70 was also confirmed as a direct target, as its expression was reduced by miR-144-3p mimics and induced by inhibitors. Decreased HSP70 levels improved granulosa cell proliferation, reduced apoptosis, decreased abnormal ovarian weight, and benefited endocrine disorders.

Anti-miR-144-3p applied in vivo increased serum testosterone, estradiol, and LH, but knockdown of HSP70 was able to minimize this increase [94]. Hence, targeting miRNAs could be a promising avenue for treating PCOS, but further research is necessary to increase knowledge about this therapeutic approach.

Besides miR-144-3p, using other microRNAs to modulate HSP activity might yield useful results as a treatment option for PCOS. Table 2 summarizes the roles of different microRNAs in regulating the activity of HSPs. However, it should be noted that limited research is available on the effect of microRNAs on the activity of HSPs in the context of PCOS.

Despite these promising findings, there are some obstacles to using HSPs therapeutically. It is challenging to ensure that the specific HSP is upregulated in the intended tissue, at the correct level/specificity, and at a suitable time together with all the necessary co-chaperones present to yield the desired results [101]. It is likely that pharmaceutical inducers may not be able to reach the desired cells or bring about the intended effects. Moreover, research combining heat therapy and exercise has still not been explored, which could potentially be promising for considering the additive benefits of both therapeutic interventions.

## 7. Future Directions

Research to date on the role of HSPs in the pathogenesis of PCOS is limited.

Further research is warranted in the following areas:

- Studies to elucidate the detailed molecular mechanisms underlying the function of HSPs in human ovarian cells, as modulating the activity of HSPs may lead to novel strategies for treating PCOS.
- Further studies focusing upon on the expression profiles and mutations of the different HSPs in ovarian cell function, as current information is limited.
- Studies need to be conducted on the therapeutic and prognostic relevance of expression profiles of HSPs. There is limited research on the involvement of HSPs in the pathogenesis of diabetes for example using PCOS as a model.
- Studies of the effects of HSPs on PCOS pathophysiology using human samples, as a key limitation is the dearth of information in this area and studies on mouse oocyte models in vitro may not simulate PCOS oocyte development.
- Adequately powered studies are needed to address the influence of HSPs on lean, overweight, and obese cohorts with and without PCOS.
- Studies in PCOS populations of differing ethnicity are needed to determine the generalizability of findings across ethnic groups.
- Studies employing targeted manipulation of HSPs to determine their potential clinical utility for treating PCOS and its related conditions.

## 8. Conclusions

The available data suggest that HSPs play a role in the pathogenesis of PCOS and its sequelae. The differing expression levels of HSPs in ovarian tissues of women with PCOS can affect cell apoptosis and proliferation, as well as follicle development. High levels of HSP70 can also increase the expression of Th17 genes, which may disrupt immune homeostasis and increase inflammation. Abnormal expression of HSPs is also found in T2D patients, and there is evidence for an association between HSPs and IR. HSPs may also play a role in the pathogenesis of obesity through inflammatory mechanisms that are amplified by the combination of PCOS and obesity. Research on therapeutic interventions for PCOS indicates that the HSP response to moderate-intensity exercise may be impaired in women with PCOS. Heat therapy, as a therapeutic intervention, increased HSP levels in PCOS subjects and improved metabolic health through HSP-mediated inhibition of inflammatory factors. Furthermore, the modulation of the activity of HSPs for the reduction of insulin resistance and the repression of HSPs through miRNAs were also shown to alleviate symptoms of PCOS. Despite this promising research, more studies using human samples are needed to define in detail the mechanisms by which HSPs impact ovarian cells, their role in the pathogenesis of PCOS and its related conditions, and their potential use as therapeutic interventions. The contradictory evidence concerning the levels of different HSPs and their specific roles in ovarian tissue most definitely requires further study.

## Figures and Tables

**Table 1:** A summary of the evidence relating to the role of HSPs in the pathogenesis of PCOS.

Authors	Year	HSPs	Aim of the Study	Main Findings
Li L et al. [11]	2016	90B1	To analyze protein expression profiles in the ovarian tissues of subjects with PCOS	HSP90B1’s expression was increased by at least 2-fold and associated with the proliferation and survival of ovarian cells
Velázquez MML et al. [38]	2013	27 and 60	To analyze HSP expression changes in bovine ovaries with cystic ovarian disease (COD) (induced by ACTH)	Induced COD caused differences in HSP protein expression
Wu G et al. [33]	2016	70	To study the correlation between HSP70 and hormones and inflammatory factors and investigate the role of HSP70 in the pathogenesis of PCOS	HSP70 showed abnormal expression in PCOS, which correlated with testosterone and inflammatory factors
Zhao K-K et al. [41]	2013	10	To determine the effect of HSP10 on apoptosis induced by testosterone in granulosa cells of mouse ovaries	Testosterone may reduce HSP10 expression in granulosa cells causing reduced Bcl-2 expression and increased Bax expression
Liu J-J et al. [43]	2010	27	To investigate the effects of HSP27 downregulation on oocyte development	Reduction in HSP27 levels improved the maturation of mouse oocytes and increased the early stage of apoptosis in oocytes
Cai L et al. [44]	2013	27	To investigate the effects of upregulation of HSP27 on oocyte development and maturation in PCOS	Upregulation of HSP27 led to inhibition of oocyte maturation in women with PCOS
Yang Y et al. [32]	2021	70	To study the correlation between HSP70 and Treg/Th17 ratio	Abnormal levels of HSP70 were correlated with Treg/Th17 imbalance, indicating that HSP70 plays a role in PCOS immunological pathogenesis

**Table 2:** A list of possible microRNAs and their role in the regulation of HSPs. These microRNAs may act as potential treatment options for modulating the activity of HSPs to yield favorable results in women with PCOS.

MicroRNA	Role in Regulation of HSPs
miR-570 [97]	miR-570 was found to affect tumor cell growth and migration by targeting the HSP chaperone network
miR-223 [98]	miR-223 can inhibit cell proliferation and increase cell apoptosis by targeting HSP 70
miR-550a-3p [99]	miR-550a-3p can have antiproliferative and proapoptotic effects in prostate and ovarian cancer cells by inhibiting HSP90AA1
miR-1 [100]	miR-1 was found to play a role in nitric oxide-induced apoptosis in osteoblasts by targeting HSP70

## Data Availability

Not applicable.
